# A Cross-sectional Survey of HIV Transmission and Behavior among Men Who Have Sex with Men in Different Areas of Inner Mongolia Autonomous Region, China

**DOI:** 10.1186/s12889-016-3809-z

**Published:** 2016-11-15

**Authors:** Lin Qu, Wenrui Wang, Yongming Gao, Jingyuan Yang, Jijiang Dai, Dawei Wang, Bo Tao

**Affiliations:** 1 Inner Mongolia Autonomous Region Center for Disease Control and Prevention, Hohhot, 010031 Inner Mongolia People’s Republic of China; 2 Baotou Center for Disease Control and Prevention, Baotou, 014030 Inner Mongolia People’s Republic of China; 3 Hohhot Center for Disease Control and Prevention, Hohhot, 010031 Inner Mongolia People’s Republic of China; 4 Inner Mongolia Autonomous Region Center for Disease Control and Prevention, No. 50 Erdos Street, Yuquan District, Inner Mongolia Autonomous Region China

**Keywords:** HIV, Risky behaviors, Men who have sex with men, Inner Mongolia Autonomous Region, Cross-sectional survey

## Abstract

**Background:**

Little research has been conducted on the human immunodeficiency virus (HIV) epidemic and the sexual intercourse habits of men who have sex with men (MSM) in crowded places, both locally and abroad. This study conducted a survey of MSM in different locales of Inner Mongolia to provide a reference for developing strategies or measures to prevent and control HIV among this understudied population.

**Methods:**

We conducted a cross-sectional survey of men aged 18 years and older at different venues popular among MSM in Inner Mongolia. Between April and July 2012, MSM volunteered to participate in this study, receive HIV/syphilis testing, and complete a questionnaire about their behavior. A total of 1611 MSM participated. Participants signed a voluntary informed consent form, completed an anonymous questionnaire and were tested for HIV and syphilis antibodies.

**Results:**

Of the 1611 MSM surveyed, 6.83 and 23.65 % had HIV and syphilis, respectively, and the co-infection rate was 3.17 %. Sociodemographic factors such as age, culture, marital status, knowledge of acquired immune deficiency syndrome (AIDS) transmission, and peer education significantly differed between venues (*P <* 0.01). MSM who were under 22 years, 23–35 years, and over 36 years primarily contacted their potential partners online, at bars/other (streetwalkers), and at public baths/parks, respectively. MSM partners found in bars, in public baths, in parks and online were primarily high school students and technical secondary school students. MSM who were streetwalkers or cross-dressing male sex workers primarily had junior middle school education levels or below. Married MSM primarily had intercourse in public baths and parks, and MSM who had intercourse in public baths and parks also reported the greatest proportions of intercourse with women (39.1 and 35.0 %, respectively). Furthermore, MSM who had intercourse in parks reported having the most anal sex with same-sex partners and unprotected intercourse in the past 6 months. Unprotected intercourse with women in the past 6 months was also common among MSM who met partners in bathhouses or online. MSM were most likely to have anal sex with other men in public baths. MSM who had intercourse in bars were the least likely to have used a condom with female partners in the past 6 months. The culture of the MSM who had frequent intercourse with streetwalkers and cross-dressing male sex workers did not predict behavior.

**Conclusion:**

This study indicated that AIDS-related risky behaviors as well as HIV and syphilis infection were associated with the different locations frequented by MSM. When developing intervention strategies for AIDS, it is better to conduct targeted health education and behavioral interventions at bars/online for MSM aged 23–35 years and at public baths/parks for MSM over 36 years. Additionally, the current survey showed that information on AIDS/sexually transmitted diseases (STDs) must be popularized to reach streetwalkers and cross-dressing male sex workers, whose mobility limits their attainment of higher levels of health education.

## Background

Although substantial efforts to prevent and control human immunodeficiency virus (HIV) infection and acquired immune deficiency syndrome (AIDS) have been implemented worldwide, the prevalence of these conditions has increased each year. According to the latest statistics, men who have sex with men (MSM) represent a major source of new HIV cases in North America and East Asia [1]. Data from the Ministry of Health of the People’s Republic of China show that the proportion of Chinese MSM with AIDS increased from 14.7 % in 2009 to 17.4 % in 2011, indicating that the prevalence of AIDS has increased rapidly among MSM [2]. A report in 2013 showed that 21.4 % of people newly diagnosed with AIDS in China were exposed to HIV through homosexual sex [3]. In China, MSM live in secrecy, and it is therefore difficult for them to have stable sexual relationships. Moreover, they tend to have a relatively large number of sexual partners (including both stable and occasional sexual partners), and they prefer practicing relatively risky sexual behaviors (e.g., anal and oral sex). Therefore, this population is a high-risk group for HIV infection.

The Inner Mongolia Autonomous Region is located in northern China. With its rapid and recent economic development, numerous farmers, herdsmen, and migrants have moved into its cities, with the fastest economic growth occurring in those areas. Homosexual sex mostly occurs among this population. As of December 31, 2012, 1392 people in this region were HIV-infected, including 269 patients and 138 reported deaths [4]. The AIDS epidemic continues to increase each year, and the Inner Mongolia Autonomous Region has particularly high rates of both sexually transmitted AIDS and syphilis among MSM and other high-risk groups. However, no data are yet available regarding the HIV infections caused by MSM who seek partners in different venues, and this lack of data impedes efforts to prevent and control HIV transmission among MSM. Accordingly, we conducted a survey among MSM between April and July 2012 to provide a reference for the development of strategies or measures to prevent and control HIV among this population in Inner Mongolia.

Few reports address HIV infection and behavior among MSM in different venues. A better understanding of the MSM who seek sex in bathhouses, bars, parks and other areas might lead to an improvement in AIDS prevention programs and intervention models. The current survey was conducted with this goal in mind.

## Methods

### Cross-sectional survey

Participant data were collected with the approval of the Medical Ethics Committee of the Centers for Disease Control and Prevention, Inner Mongolia Autonomous Region. The target population was men aged 18 years and older from the Inner Mongolia Autonomous Region who had had oral or anal sex with other men within the past year and volunteered to receive HIV and syphilis testing.

### Data collection

Participants signed a voluntary informed consent form and then completed an anonymous questionnaire. The questionnaire consisted of two sections: the first section pertained to sociodemographic information, knowledge of AIDS, sexual behavior, condom use, and previous AIDS testing, and the second section concerned information about AIDS and syphilis detection. These data were collected by the local Centers for Disease Control and Prevention. Each questionnaire had a unique number.

### Site survey

Training: The Inner Mongolia Autonomous Region Center for Disease Control and Prevention of the trained core MSM work-group members in Hohhot and Baotou to ensure that they had a clear understanding of the purpose of the study and were fully inspired, thereby ensuring the authenticity of the sample. Next, a comprehensive peer intervention was conducted among the MSM population.

Grouping: The locations MSM use to seek sexual partners were classified into five groups: bars (e.g., bars, dance halls, tea rooms, clubs); public baths (e.g., public baths, saunas, foot massage and massage parlors); parks (parks, public toilets, lawns, and squares); the Internet; and other (solicitation of streetwalkers and cross-dressing male sex workers).

Understanding of basic AIDS facts: The percentage of participants who knew basic facts about AIDS was calculated using the “AIDS Monitoring and Evaluation Framework of China (trial use)”. Respondents who correctly answered six or more questions were considered to have basic knowledge of AIDS. The questions included the following eight items: 1. Can a person tell that another person is infected with HIV based on appearance? 2. Can a person receive HIV from a mosquito bite? 3. Can a person receive HIV by eating with a person infected with HIV? 4. Can a person receive HIV via an HIV-infected blood transfusion? 5. Can a person receive HIV by sharing syringes with a person infected with HIV? 6. Can a baby born to an HIV-positive mother be infected with HIV? 7. Can the correct use of condoms reduce the spread of AIDS? and 8. Can having sex with only one partner reduce the spread of AIDS?

Referrals to preferred sexually transmitted disease (STD) clinic services were offered. Referrals to online information and consultations regarding AIDS were conducted through two websites: “Hohhot Sunshine Beyond the Frontier” and “Baotou Online Gay Platform.”

### Laboratory testing

Five mL of blood was collected from each respondent to test for HIV and syphilis antibodies. Respondents who were HIV antibody-positive at the preliminary screening also received a confirmatory test. Preliminary HIV antibody screening was performed using reagents produced by Zhuhai Livzon Diagnostics Inc., and the confirmatory Western blots were performed using products by Beijing Wantai Bio-Pharmaceutical Co., Ltd.. Syphilis antibody screening was conducted via an enzyme-linked immunosorbent assay (ELISA), using reagents produced by Zhuhai Livzon Diagnostics Inc., and confirmatory syphilis testing was implemented via toluidine red unheated serum test (TRUST) using reagents produced by Beijing Wantai Bio-Pharmaceutical Co., Ltd.

### Statistical analyses

The data were entered using EpiData 3.10 (Odense, Denmark) and analyzed using SPSS 15.0. All data were expressed as the mean ± standard deviation (SD). *P*-values < 0.05 were considered significant. Descriptive analyses, univariate *χ* ^2^ tests, and unordered multinomial logistic regression were employed for statistical analysis.

## Results

### General information

A total of 1611 MSM participated in this study. Of these participants, 1513 were of Han ethnicity, and 98 were of other ethnicities; 1390 (86.28 %) were from the Inner Mongolia Autonomous Region, and 221 (13.72 %) were from other provinces. Participants’ ages ranged from 18 to 78 years (mean = 33.00 ± 9.4 years); 905 (56.18 %) were unmarried, 549 (34.08 %) were married, one (0.06 %) was cohabiting, and 156 (9.68 %) were divorced or widowed. Additionally, 390 (24.21 %) had a college degree or higher, 771 (47.86 %) had a senior high school or technical secondary school level of education, and 450 (27.93 %) had a junior middle school education or lower.

### Meeting places

To meet sexual partners, 158 (9.81 %) respondents preferred bars, 373 (23.15 %) preferred public baths, 928 (57.61 %) preferred parks, 93 (5.77 %) preferred the Internet, and 59 (3.66 %) preferred solicitation of streetwalkers or cross-dressing prostitutes.

#### Meeting places by age

A significant difference emerged between age groups regarding the choice of where to meet potential partners (Table 1; *χ* ^2^ = 57.97, *P* < 0.01). Respondents who were ≤ 22 years old mostly preferred the Internet; those who were 23–35 years old mostly preferred visiting bars or soliciting streetwalkers or cross-dresses prostitutes; and those ≥ 36 years old mostly preferred bathhouses and parks (Table 2).

**Table 1:** Univariate chi-square analysis of MSM by meeting place

Demographics		Number of respondents	Meeting place					*χ* ^2^	*P*
			Bars	Public baths	Parks	Internet	Others		
Age (years)		1611						57.97	<0.01
	≤22	183 (11.36)	27	19	113	19	5		
	23–35	820 (50.90)	91	190	442	57	40		
	≥36	608 (37.74)	40	164	373	17	14		
Education level		1611						120.35	<0.01
	Junior high school or lower	450 (27.93)	22	79	292	11	46		
	Senior high school or technical secondary school	390 (24.21)	45	108	203	33	1		
	College degree or higher	771 (47.86)	91	186	433	49	12		
Marital status		1611						49.8	<0.01
	Unmarried	905 (56.18)	111	179	513	62	40		
	Married	550 (34.14)	36	155	326	27	6		
	Divorced or widowed	156 (9.68)	11	39	89	4	13		
Registered permanent residence		1611						2.55	0.636
	Inner Mongolia Autonomous Region	1390 (86.28)	140	319	805	77	49		
	Other provinces	221 (13.72)	18	54	123	16	10		
Basic knowledge of AIDS		1611						55.22	<0.01
	No	160 (9.93)	8	30	92	8	22		
	Yes	1451 (90.07)	150	343	836	85	37		
Homosexual anal sex in the past 6 months		1611						44.33	<0.01
	No	240 (14.90)	17	28	183	11	1		
	Yes	1371 (85.10)	141	345	744	82	59		
Condom use during most recent homosexual anal sex		1371						29.25	<0.01
	No	241 (17.58)	22	48	163	8	0		
	Yes	1130 (82.42)	119	297	581	74	59		
Frequency of homosexual anal sex in the past week		1368						131.38	<0.01
	0	56 (4.09)	3	9	34	7	3		
	1	717 (52.41)	71	156	454	27	9		
	2	401 (29.31)	45	131	172	34	19		
	3	129 (9.43)	18	30	56	10	15		
	4	53 (3.88)	2	15	22	3	11		
	5	12 (0.88)	2	1	6	1	2		
Frequency of condom use for homosexual anal sex in the past 6 months		1371						13.27	0.103
	Never	30 (2.19)	3	3	22	1	1		
	Occasionally	738 (53.83)	66	183	420	39	30		
	Always	603 (43.98)	72	158	302	42	29		
Commercial homosexual sex in the past 6 months		1371						442.1	<0.01
	No	1288 (93.95)	134	342	713	81	18		
	Yes	83 (6.05)	7	3	31	1	41		
Heterosexual sex in the past 6 months		1611						46.99	<0.01
	No	1086 (67.41)	123	227	603	78	55		
	Yes	525 (32.59)	35	146	325	15	4		
Frequency of condom use for heterosexual sex in the past 6 months		525						18.81	0.016
	Never	183 (34.86)	15	36	128	2	2		
	Occasionally	247 (47.04)	17	79	143	7	1		
	Always	95 (18.10)	3	31	54	6	1		
Condom use at most recent heterosexual sex		525						13.52	<0.01
	No	289 (55.05)	22	65	195	5	2		
	Yes	236 (44.95)	13	81	130	10	2		
Condom use promotion and distribution/AIDS counseling and testing		1611						30	<0.01
	No	34 (2.11)	2	3	18	9	2		
	Yes	1577 (97.89)	156	370	910	84	57		
Peer education		1611						120.04	<0.01
	No	365 (22.66)	29	89	162	45	40		
	Yes	1246 (77.34)	129	284	766	48	19		
HIV or syphilis infection		1611							
	HIV-positive	110 (6.83)	8	31	62	7	2	3.261	0.515
	Syphilis-positive	381 (23.65)	37	86	226	14	18	5.675	0.225
	Both	51 (3.17)	2	15	29	3	2	2.767	0.598

**Table 2:** Ages of MSM who prefer different meeting places

Places/Composition/Age	≤22	23–35	≥36
Bars	17.10 %	57.60 %	25.30 %
Public baths	5.10 %	50.90 %	44.00 %
Parks	12.20 %	47.60 %	40.20 %
Internet	20.40 %	61.30 %	18.30 %
Other	8.50 %	67.80 %	23.70 %

#### Meeting places by education level

Education level had a significant effect on choice of meeting place (*χ* ^2^ = 120.35, *P* < 0.01). Respondents with a senior high school, technical secondary school or higher level of education mostly preferred the Internet and bars, whereas those with a junior high school education or lower preferred parks, forming the second largest group of participants. Respondents with a junior high school education or lower also preferred soliciting streetwalkers and cross-dressing prostitutes.

#### Meeting places by marital status

Preferred meeting places differed significantly by marital status (*χ* ^2^ = 49.8, *P* < 0.01). Unmarried MSM primarily preferred bars; married MSM primarily preferred public baths and parks; and divorced or widowed MSM primarily preferred soliciting streetwalkers and cross-dressing male sex workers.

### Knowledge of AIDS

Of the MSM who completed the questionnaire, 1451 (90.07 %) had basic knowledge of AIDS. A significant difference was found with regard to meeting place based on level of education (*χ* ^2^ = 55.22, *P* < 0.01). The highest proportion of those with basic AIDS knowledge was observed among the respondents who preferred bars, whereas the lowest (62.71 %) occurred among those who preferred to solicit streetwalkers and cross-dressing prostitutes.

### Homosexual sex

Overall, 85.10 % (1371/1611) of the respondents reported having had anal sex with a male partner in the past 6 months; of those, 2.19 % (30/1371) reported having unprotected anal sex, and 43.98 % (603/1371) reported consistently using condoms. The occurrence of homosexual anal sex in the past 6 months differed significantly by meeting place (*χ* ^2^ = 44.33, *P* < 0.01); however, the frequency of condom use for homosexual anal sex did not (*χ* ^2^ = 13.27, *P* > 0.05). The frequency of homosexual anal sex in the past week differed significantly by meeting place (*χ* ^2^ = 131.38, *P* < 0.01). Those who preferred parks had the highest proportion of participants who reported having homosexual anal sex once or less in the previous week, whereas respondents who preferred the Internet or soliciting streetwalkers and cross-dressing prostitutes reported having had anal sex 2 or 3 times in the previous week. The use of condoms during recent homosexual anal sexual encounters differed significantly by meeting place (*χ* ^2^ = 29.25, *P* < 0.01); respondents who preferred parks were the most likely to have unprotected anal sex. The occurrence of commercial homosexual sex in the past 6 months differed significantly by meeting place (*χ* ^2^ = 442.10, *P* < 0.01); respondents who preferred soliciting streetwalkers and cross-dressing prostitutes were more likely to have had commercial sex.

### Heterosexual sex

**Fig. 1:**
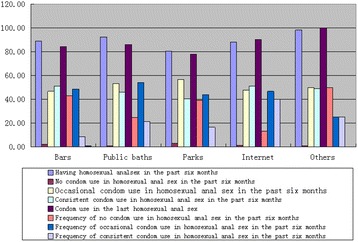
MSM behaviors by meeting place

Overall, 32.5 % (525/1611) of the respondents reported having heterosexual sex in the past 6 months; of those, 34.9 % (183/525) had unprotected heterosexual sex, whereas 18.1 % (95/525) consistently used condoms. The proportion of respondents who had heterosexual sex in the past 6 months differed significantly by meeting place (*χ* ^2^ = 46.99, *P* < 0.01); respondents who preferred public baths (39.1 %), followed by those who preferred parks (35.0 %), were the most likely to have had heterosexual sex. The frequency of condom use also differed by meeting place (*χ* ^2^ = 18.81, *P* < 0.05). Those who preferred parks, followed by those who preferred bars, were the most likely to have had unprotected sex. Respondents who preferred public baths were the most likely to occasionally use condoms, whereas those who consistently used condoms were the most likely to prefer the Internet (Fig. 1).

### Peer education

Overall, 1246 (77.34 %) respondents had received peer education in the previous year. The receipt of peer education differed significantly by meeting place (*χ* ^2^ = 120.04, *P* < 0.01). MSM who preferred soliciting streetwalkers and cross-dressing prostitutes, followed by those who preferred the Internet, were the least likely to have received peer education.

### HIV and syphilis prevalence

Of the 1611 MSM studied, 381 (23.65 %) tested positive for syphilis, 110 (6.83 %) tested positive for HIV, and 51 (3.17 %) tested positive for both (Table 1).

### Multivariate logistic regression analysis

We conducted a logistic regression analysis of the different places that MSM sought sexual partners to determine the factors most predictive of behavior. These findings could provide insight into the next steps for interventions, health education and health promotion.

To remove the influence of confounding variables, the logistic regression analysis included predictors such as sociodemographic information, knowledge of AIDS, and sexual behavior characteristics. The results revealed that age, education level, marital status, knowledge of AIDS, frequency of homosexual anal sex in the previous week, heterosexual sex in the previous 6 months, commercial homosexual anal sex in the previous 6 months, and peer education predicted MSM’s preferences for meeting places (*P* < 0.05). The influence of age on preference for public baths was 0.097 times the influence of age on other meeting places. A high level of cultural beliefs and knowledge of AIDS were associated with less anal sex among those who solicited commercial sex. The frequency of homosexual anal sex in the previous week showed the largest influence on preferences for public baths (OR = 1.582, 95 % CIs = 2.132–1.172). Heterosexual sex in the previous 6 months was associated with a 3.63- and 3.17-times odds of preferring public baths and parks, respectively, compared with preferring other places (Table 3).

**Table 3:** Multivariate logistic analysis of MSM by meeting place

Influence factor	Dependent variable	$\widehat{\beta}$	*S.E.*	Wald *χ* ^*2*^	*P*	OR	OR (95 % CIs)	
							OR_L_	OR_U_
Age	Bars	−0.994	0.835	1.415	0.234	0.370	0.072	1.903
	Public baths	−2.330	0.886	6.914	0.009	0.097	0.017	0.553
	Parks	0.033	0.702	0.002	0.963	1.034	0.261	4.095
	Internet	−1.117	0.929	1.445	0.229	0.327	0.053	2.023
Marital status	Bars	−0.536	0.202	7.048	0.008	0.585	0.394	0.869
	Public baths	−0.165	0.179	0.848	0.357	0.848	0.598	1.204
	Parks	−0.322	0.171	3.543	0.060	0.724	0.518	1.013
	Internet	−0.452	0.228	3.914	0.048	0.636	0.407	0.996
Basic knowledge	Bars	1.738	0.661	6.908	0.009	5.686	1.556	20.784
	Public baths	1.539	0.572	7.251	0.007	4.660	1.520	14.286
	Parks	1.183	0.547	4.673	0.031	3.265	1.117	9.547
	Internet	1.846	0.683	7.301	0.007	6.332	1.660	24.150
Education level	Bars	1.761	0.405	18.876	<0.001	5.817	2.629	12.873
	Public baths	1.809	0.399	20.580	<0.001	6.103	2.794	13.334
	Parks	1.529	0.391	15.256	<0.001	4.612	2.142	9.932
	Internet	1.997	0.424	22.229	<0.001	7.366	3.212	16.896
Commercial homosexual anal sex in the past six months	Bars	−4.444	0.651	46.552	<0.001	0.012	0.003	0.042
	Public baths	−6.031	0.778	60.111	<0.001	0.002	<0.001	0.011
	Parks	−4.465	0.551	65.610	<0.001	0.012	0.004	0.034
	Internet	−5.829	1.143	26.027	<0.001	0.003	<0.001	0.028
Heterosexual sex in the past six months	Bars	0.321	0.623	0.266	0.606	1.379	0.406	4.680
	Public baths	1.288	0.590	4.770	0.029	3.626	1.141	11.518
	Parks	1.154	0.582	3.935	0.047	3.170	1.014	9.911
	Internet	0.287	0.658	0.190	0.663	1.332	0.367	4.832
Peer education	Bars	2.150	0.534	16.195	<0.001	8.581	3.012	24.444
	Public baths	1.789	0.508	12.400	<0.001	5.984	2.211	16.197
	Parks	2.115	0.494	18.330	<0.001	8.286	3.147	21.816
	Internet	0.508	0.549	0.857	0.355	1.662	0.567	4.869

## Discussion

Previous studies have performed only single-factor analyses on the prevalence of AIDS/STDs and the percentage of MSM with basic knowledge of AIDS transmission [5–39]. Most of these studies investigated the prevalence of AIDS/STDs among MSM who sought sex on the Internet [12–16, 18–23, 27–29]. This study was the first to analyze and compare multiple predictors (e.g., age and marital status) among MSM seeking sex at different venues, and the findings could enable the development of more effective intervention strategies for MSM based on their preferred meeting place. The results of this study are thus more applicable than those of previous studies.

Most MSM from the Inner Mongolia Autonomous Region of China in our sample were between 23 and 35 years old; this finding is consistent with the survey results of Luo et al. of 259 MSM in Hangzhou (74.5 %) [40] but inconsistent with those of Hakim et al., who found that 35.9 % of 603 MSM in Abidjan were between 23 and 35 years old [41].

The present study found that MSM sought sex in parks most often, followed by public baths and then bars. MSM who preferred meeting partners in parks and public baths were mostly middle-aged married men with lower levels of education; those who sought sex in bars were mostly young, highly educated, and unmarried; and those who sought sex on the Internet were mostly young, highly educated and unmarried students. Furthermore, MSM who sought sex by soliciting prostitutes were primarily young, less educated, and divorced; many were streetwalking and cross-dressing male sex workers. These results differ from previous findings, which showed that MSM in China who pursued sex via other means mostly sought partners through introductions from friends. These MSM were highly educated, of a high social status, and unmarried [42–44]. This difference in results might be primarily because of the difference in the volunteers’ social circles and the resulting difference in respondents. In general, people are more likely to trust those of a similar cultural, economic, and social background [44].

This study found that a high percentage of participants were familiar with basic facts about AIDS transmission (90.07 %, 1451/1611), which is consistent with the findings of Luo et al. (90 %) [40]. The percentage of participants with basic knowledge of AIDS transmission differed by their preferred meeting place. Respondents who preferred bars, followed by those who preferred public baths, the Internet, and parks, were the most likely to have basic AIDS knowledge, whereas those who preferred soliciting streetwalkers and cross-dressing prostitutes were the least likely (<80 %). A separation between knowledge and behavior was observed among MSM respondents according to meeting place. This finding suggests that although current interventions focus on increasing the public’s knowledge of AIDS (MSM have an accurate knowledge of AIDS overall), it is difficult to change MSM’s high-risk behaviors, such as having multiple sexual partners, a high frequency of sex, and unprotected sex [45, 46]. On the other hand, although the role of condoms in disease prevention has been emphasized, this focus has deterred condom use among many people because they consider condoms to be synonymous with disease [47]. Therefore, the root causes of not using or not consistently using condoms must be identified with regard to condom promotion to ensure that effective prevention and treatment measures can be achieved.

MSM who preferred soliciting streetwalkers and cross-dressing prostitutes, followed by those preferring the Internet, were the most likely to report receiving peer education. This finding suggests that social network platforms (e.g., QQ, forums, and microblogs) could be better used by local AIDS knowledge publicity campaigns to ensure the dissemination of effective health education.

Sex with multiple partners and unprotected sex are most likely to occur among MSM at public baths [48, 49]. The present results revealed that having anal sex multiple times was also more likely to occur at bathhouses. MSM who seek sex at parks and public baths were more likely to be married and to have had unprotected heterosexual sex in the previous 6 months, in their most recent heterosexual sex act, and in their most recent homosexual sex act. This finding might be related to their older age, lower education level, marital status, their method of passively seeking homosexual sexual partners, or their low self-protection awareness and abilities [44]. The individuals in this group could easily infect their spouses and thus represent a “bridge” from the MSM population to the general population of HIV and syphilis transmission [50]. In addition, this finding suggests that MSM who seek sex at parks and public baths are a high-risk population for HIV and syphilis infection.

MSM who seek sex in other ways accounted for only 3.66 % (59/1611) of the sample; these participants primarily sought sex via streetwalkers and cross-dressing prostitutes. This result differs from the findings of a previous study, which found that MSM mostly sought partners through introductions by friends [43]. According to our survey results, this group was less educated, knew fewer basic facts about AIDS transmission and was more likely to have paid for homosexual sex. Moreover, they had poorer economic conditions, were highly sexually active, and usually assumed a subordinate position in intercourse; all of these factors make them more likely to demonstrate high-risk behaviors. Therefore, we recommend that public education programs promote knowledge of AIDS prevention and treatment in addition to providing free condoms [51].

MSM preferences for meeting places were related to age, education level, marital status, knowledge of AIDS, frequency of homosexual anal sex in the past week, heterosexual sex in the past 6 months, commercial homosexual anal sex in the past 6 months, and peer education (*P* < 0.05). Young MSM were more likely to prefer bars and the Internet, whereas middle-aged men preferred public baths and parks; this difference might be related to the participants’ cultural and recreational environments. The association between the frequency of homosexual anal sex in the past week and preferred meeting places was strongest for public baths (OR = 1.582, 95 % CIs = 2.132–1.172), indicating that public baths were the primary place that the MSM surveyed in this study chose to seek sex. MSM who sought sex via the Internet and bars were less likely to engage in heterosexual sex.

A total of 1611 MSM received syphilis and HIV testing in this study. Of these participants, 381 (23.65 %) tested positive for syphilis, 110 (6.83 %) tested positive for HIV, and 51 (3.17 %) tested positive for both; these rates are higher than those reported among MSM living in 61 Chinese cities (HIV = 4.9 %; syphilis = 11.8 %) [52] and higher than the HIV incidence estimated among Chinese MSM in 2011 (6.3 %) [40, 53, 54]. Syphilis can promote the spread of AIDS through ulcerations, and a high syphilis incidence enables HIV to spread rapidly. Therefore, STD diagnosis and treatment services among MSM should be strengthened and standardized, syphilis treatment referrals should be improved, and HIV screening should be conducted among patients at STD clinics to enhance early diagnosis and treatment.

In summary, MSM generally show high-risk sexual behaviors. Targeted health education and behavioral interventions should be conducted among MSM based on their meeting places to prevent and control the spread of HIV. Although this study analyzed only monitoring data from the Inner Mongolia Autonomous Region of China in 2012, the results revealed certain patterns that could provide a strong basis for conducting interventions among MSM at different meeting places in this region.

This survey has some limitations. Because snowball sampling was employed instead of common statistical sampling methods, the sample was not fully representative of the target population. Specifically, because of the discrete nature of the MSM population, inactive and elderly MSM were not included in sufficient numbers. The behavioral characteristics studied might also be biased. Access to the Internet was high, but not ideal, in the survey area in 2012. Furthermore, online communication primarily occurred via QQ. Because the community team was not experienced with online surveys, only a small number of MSM who sought sex via the Internet were included, which might have led to an underestimation of the actual results.

## Conclusions

The results of this study indicate that in 2012, there were high rates of HIV and syphilis as well as a high level of knowledge of the basic facts of AIDS transmission among MSM who sought sex at different places. Multiple sexual contacts and unprotected sex were most likely to occur among MSM in public baths. A separation between knowledge and behavior was observed among MSM who preferred different meeting places. High-risk sexual behaviors were observed in most MSM. Targeted health education and behavioral interventions should be implemented based on the preferred meeting places of MSM to better prevent and control HIV and AIDS.

## Abbreviations

AIDS: Acquired immune deficiency syndrome
HIV: Human immunodeficiency virus
MSM: Men who have sex with men
STD: Sexually transmitted disease

## References

[1] Beyrer C, Baral SD, van Griensven F, Goodreau SM, Chariyalertsak S, Wirtz AL (2012). Global epidemiology of HIV infection in men who have sex with men. Lancet.

[2] Li H-M, Peng R-R, Li J, Yin YP, Wang B, Cohen MS (2011). HIV incidence among men who have sex with men in China: a meta-analysis of published studies. PLoS One.

[3] NCAIDS NCSTD, China CDC (2014). Update on the AIDS/STD epidemic in China and main response in control and prevention in December, 2013. Chin J AIDS STD.

[4] Qu L, Gao YM, Li X (2013). AIDS epidemic among men who have sex with men in Inner Mongolia Autonomous Region, China from 2008 to 2012. Chronic Pathematol J.

[5] Liu MH, Zhang BC, Li JF (2006). Analysis of sociological and related factors in men who have sex with men. Chin J AIDS STD.

[6] Li DM, Ge L, Wang L (2014). Variation tendency in AIDS and related behaviors among men who have sex with men in China from 2010 to 2013. Chin J Epidemiol.

[7] Zhu JL, Zhang HB, Wu HH (2007). High risk sexual behavior and HIV/STD infection rate among 122 MSM from students. Chin J AIDS STD Prev Control.

[8] Ding XB, Zhang W, Feng LG (2011). AIDS epidemic situation and prevention strategies in Chongqing, China. J Trop Med.

[9] Chen Q, Li Y, Su X, Hao M, Lu H, He X (2014). Epidemiological analysis on recent infected HIV-1 patients among newly reported HIV cases in Beijing, from 2009 to 2011. Chin J Epidemiol.

[10] Zhang BC, Liu DC, Li JF (2001). Study on HIV/STD high risk behavior and its factors among men who have sex with men in China. Chin J AIDS STD Prev Control.

[11] Glick SN, Morris M, Foxman B, Aral SO, Manhart LE, Holmes KK (2012). A comparison of sexual behavior patterns among men who have sex with men and heterosexual men and women. J Acquir Immune Defic Syndr.

[12] Bolding G, Davis M, Hart G, Sherr L, Elford J (2005). Gay men who look for sex on the internet: is there more HIV/STI risk with online partners?. AIDS.

[13] Benotsch EG, Kalichman S, Cage M (2002). Men who have met sex partners via the internet: prevalence, predictors, and implications for HIV prevention. Arch Sex Behav.

[14] Garofalo R, Herrick A, Mustanski BS, Donenberg GR (2007). Tip of the Iceberg: young men who have sex with men, the internet, and HIV risk. Am J Public Health.

[15] Liau A, Millett G, Marks G (2006). Meta-analytic examination of online sex-seeking and sexual risk behavior among men who have sex with men. Sex Transm Dis.

[16] Chiasson MA, Hirshfield S, Remien RH, Humberstone M, Wong T, Wolitski RJ (2007). A comparison of on-line and off-line sexual risk in men who have sex with men: an event-based on-line survey. J Acquir Immune Defic Syndr.

[17] Horvath KJ, Rosser BR, Remafedi G (2008). Sexual risk taking among young internet-using men who have sex with men. Am J Public Health.

[18] Blackwell CW (2008). Men who have sex with men and recruit bareback sex partners on the internet: implications for STI and HIV prevention and client education. Am J Mens Health.

[19] Hooper S, Rosser BR, Horvath KJ, Oakes JM, Danilenko G, Men’s Internet Sex II (MINTS-II) Team (2008). An online needs assessment of a virtual community: what men who use the internet to seek sex with men want in Internet-based HIV prevention. AIDS Behav.

[20] Mustanski B, Lyons T, Garcia SC (2011). Internet use and sexual health of young men who have sex with men: a mixed-methods study. Arch Sex Behav.

[21] Carballo-Diéguez A, Miner M, Dolezal C, Rosser BR, Jacoby S (2006). Sexual negotiation, HIV-status disclosure, and sexual risk behavior among Latino men who use the internet to seek sex with other men. Arch Sex Behav.

[22] Wohlfeiler D, Potterat JJ (2005). Using gay men’s sexual networks to reduce sexually transmitted disease (STD)/human immunodeficiency virus (HIV) transmission. Sex Transm Dis.

[23] Rietmeijer CA, Bull SS, McFarlane M, Patnaik JL, Douglas JM (2003). Risks and benefits of the internet for populations at risk for sexually transmitted infections (STIs): results of an STI clinic survey. Sex Transm Dis.

[24] Baral S, Sifakis F, Cleghorn F, Beyrer C (2007). Elevated risk for HIV infection among men who have sex with men in low- and middle-income countries 2000-2006: a systematic review. PLoS Med.

[25] Baral S, Adams D, Lebona J, Kaibe B, Letsie P, Tshehlo R (2011). A cross-sectional assessment of population demographics, HIV risks and human rights contexts among men who have sex with men in Lesotho. J Int AIDS Soc.

[26] Baral S, Trapence G, Motimedi F, Umar E, Iipinge S, Dausab F (2009). HIV prevalence, risks for HIV infection, and human rights among men who have sex with men (MSM) in Malawi, Namibia, and Botswana. PLoS One.

[27] Henry E, Yomb Y, Fugon L, Spire B (2012). The use of the internet in male sexual encounters by men who have sex with men in Cameroon. Digit Cult Educ.

[28] Ybarra ML, Kiwanuka J, Emenyonu N, Bangsberg DR (2006). Internet use among Ugandan adolescents: implications for HIV intervention. PLoS Med.

[29] Borzekowski DL, Fobil JN, Asante KO (2006). Online access by adolescents in Accra: Ghanaian teens’ use of the internet for health information. Dev Psychol.

[30] Magnani R, Sabin K, Saidel T, Heckathorn D (2005). Review of sampling hard-to-reach and hidden populations for HIV surveillance. AIDS.

[31] Fay H, Baral SD, Trapence G, Motimedi F, Umar E, Iipinge S (2011). Stigma, health care access, and HIV knowledge among men who have sex with men in Malawi, Namibia, and Botswana. AIDS Behav.

[32] Kroenke K, Spitzer RL, Williams JB (2001). The PHQ-9: validity of a brief depression severity measure. J Gen Intern Med.

[33] Heckathorn DD (2007). Extensions of respondent-driven sampling: analyzing continuous variables and controlling for differential recruitment. Sociol Methodol.

[34] Ybarra ML, Bull SS (2007). Current trends in internet- and cell phone-based HIV prevention and intervention programs. Curr HIV/AIDS Rep.

[35] McFarlane M, Bull SS, Rietmeijer CA (2002). Young adults on the internet: risk behaviors for sexually transmitted diseases and HIV (1). J Adolesc Health.

[36] Mustanski BS, Newcomb ME, Du Bois SN, Garcia SC, Grov C (2011). HIV in young men who have sex with men: a review of epidemiology, risk and protective factors, and interventions. J Sex Res.

[37] Horvath KJ, Oakes JM, Rosser BR (2008). Sexual negotiation and HIV serodisclosure among men who have sex with men with their online and offline partners. J Urban Health.

[38] Lansky A, Drake A, Wejnert C, Pham H, Cribbin M, Heckathorn DD (2012). Assessing the assumptions of respondent-driven sampling in the national HIV Behavioral Surveillance System among injecting drug users. Open AIDS J.

[39] Wejnert C (2009). An empirical test of respondent-driven sampling: point estimates, variance, degree measures, and out-of-equilibrium data. Sociol Methodol.

[40] Luo Y, Zhu CY, Chen SC (2015). Risk factors for HIV and syphilis infection among male sex workers who have sex with men: a cross sectional study in Hangzhou, China. BMJ Open.

[41] Hakim AJ, Aho J, Semde G, Diarrassouba M, Ehoussou K, Vuylsteke B (2015). The epidemiology of HIV and prevention needs of men who have sex with men in Abidjan, Cote d’Ivoire. PLoS One.

[42] Xu J (2010). Prevalence of HIV infection and the risk factors among MSM in 4 cities in China. J Prev Med.

[43] Tang HL, Zhang DP, Wu YH, Zhang J, Wang L, Lv F (2007). Study on the patterns of sexual contact and behavioral features of men who have sex with men. Chin J Epidemiol.

[44] Yi W, Xu J, Li ZJ (2012). HIV and syphilis infection and related behavior characteristics among MSM in different places to find sexual partners. Chin J AIDS STD.

[45] Zeng G, Xiao Y, Xu P, Feng N, Jin CR, Lü F (2009). [Evaluation of effect of community-based HIV/AIDS interventions among men who have sex with men in eighteen cities, China. Zhonghua Yu Fang Yi Xue Za Zhi.

[46] Macus U, Voss L, Kollan C (2006). HIV incidence increasing in MSM in Germany: factors infection dynamics. J Homosex.

[47] Cao NX, Xing HM (2009). AIDS interventions among MSM in China and some reflections. Chin J AIDS STD.

[48] Binson D, Pollack LM, Blair J, Woods WJ (2010). HIV transmission risk at a gay bathhouse. J Sex Res.

[49] Bingham TA, Secura GM, Behel SK, Bunch JG, Simon PA, MacKellar DA (2008). HIV risk factors reported by two samples of male bathhouse attendees in Los Angeles, California, 2001-2002. Sex Transm Dis.

[50] Ni ZM, Li J, Xu DG (2011). An investigation on the infection rate of syphilis and HIV among MSM population from public bath. Zhejiang J Prev Med.

[51] Yi W, Xu J, Li ZJ (2011). Relationship between knowledge of AIDS prevention and treatment and demographic characteristics among men who have sex with men. Pract Prev Med.

[52] Wu Z, Xu J, Liu E, Mao Y, Xiao Y, Sun X (2013). HIV and syphilis prevalence among men who have sex with men: a cross-sectional survey of 61 cities in China. Clin Infect Dis.

[53] Li X, Lu H, Cox C, Zhao Y, Zia D, Sun Y, et al. Changing respondent-driven sampling surveys from 2009 to 2011. Biomed Res Int. 2014;2014:563517. 10.1155/2014/563517 PMC3918367 24575408

[54] Zhang L, Chow EP, Jing J, Zhuang X, Li X, He M (2013). HIV prevalence in China: integration of surveillance data and a systematic review. Lancet Infect Dis.

